# US drug overdose deaths involving stimulants without opioids continued to rise in 2024

**DOI:** 10.1016/j.abrep.2026.100743

**Published:** 2026-08-27

**Authors:** Yanwei Jin, Zongbo Li, Qiyou Wu, Brandon D.L. Marshall, Erika L. Crable, Xiao Zang

**Affiliations:** a Division of Biostatistics & Health Data Science, School of Public Health, University of Minnesota, Minneapolis, MN, USA; b Division of Health Policy and Management, School of Public Health, University of Minnesota, Minneapolis, MN, USA; c Department of Epidemiology, School of Public Health, Brown University, Providence, RI, USA; d Department of Health Policy and Management, Fielding School of Public Health, University of California Los Angeles, Los Angeles, CA, USA; e University of California Los Angeles Center for Health Policy Research, Los Angeles, CA, USA

**Keywords:** Drug overdose deaths, Stimulants without opioids, Cocaine, Methamphetamine, Temporal trends, Demographic disparities

## Abstract

**Background:**

After two decades of persistent growth, US drug overdose deaths (DODs) declined sharply in 2024, largely driven by reductions in fentanyl-involved mortality. However, trends in DODs involving stimulants without opioids remain less characterized and may follow distinct patterns.

**Methods:**

We analyzed aggregated data on unintentional DODs from 2018 to 2024 using the CDC data. Age-adjusted DOD rates were calculated using the 2000 US standard population. Joinpoint regression was used to identify significant temporal changes in mortality trends across substance categories. We examined percent changes in rates from 2023 to 2024 for cocaine and methamphetamine DODs without opioids, stratified by race/ethnicity, age group, and US census region.

**Results:**

Nationally, DOD rates declined by 27.9% from their 2022 peak to 2024, paralleling reductions in fentanyl-involved deaths. In contrast, rates of DODs involving stimulants without opioids increased slightly (cocaine: +1.7%; methamphetamine: +2.4%) from 2023 to 2024. Increases were observed nationally among White populations for cocaine without opioids and among Black, American Indian/Alaska Native (AIAN), and 55+ populations for methamphetamine without opioids. The Northeast experienced the largest increases for stimulant-without-opioid deaths, while the Midwest showed pronounced racial/ethnic divergence, including increases among White populations but substantial declines among Hispanic populations. Methamphetamine-without-opioid DODs rose markedly among AIAN populations in some regions.

**Conclusion:**

These findings highlight an evolving overdose crisis and underscore the need for prevention strategies that extend beyond opioid-focused interventions to address stimulant-specific harms.

## Highlights

• US drug overdose deaths (DODs) fell 28% by 2024, driven by a drop in fentanyl DODs.
• DODs from stimulants without opioids rose despite the overall DOD decline.
• Methamphetamine without opioids DODs rose among Black, AIAN, and older populations.
• Regional patterns vary: the Northeast saw the largest rise in stimulant-only deaths.
• Prevention must expand beyond opioids to address stimulant-specific health harms.

## 1 Introduction

Between 2003 and 2022, the United States (US) experienced a persistent rise in drug overdose deaths (DODs), reaching a peak in 2022 with nearly 108,000 deaths recorded, 76% of which involved opioids and 68% synthetic opioids (other than methadone) (Garnett & Miniño, 2024). The drug overdose epidemic recently evolved into a ‘fourth wave’ characterized by polysubstance use involving stimulants and fentanyl (Kline et al., 2023). However, US Centers for Disease Control and Prevention (CDC) data indicate a record 26% decline (compared to the 2022 peak) in drug overdose mortality in 2024 (CDC/National Center for Health Statistics, 2025). The DOD rates declined across all sex, age and racial/ethnic groups, with the largest decreases observed among younger individuals (aged 34 and under) and non-Hispanic Black populations, alongside a greater (36%) reduction in deaths involving synthetic opioids than overall drug overdose deaths (Garnett & Miniño, 2026). A recent analysis of this downturn found that national DOD rates began to fall in August 2023, though the trends were heterogeneous: opioid-related deaths declined faster than those involving stimulants, the peak occurred later in the West region, and reductions were slower among older adults and racial/ethnic minority populations (Post et al., 2025). Critically, however, prior studies have not stratified analyses by specific drug combinations, particularly distinguishing between stimulant-involved DODs with and without opioids. This distinction is vital because the aggregate decline, largely driven by reductions in fentanyl fatalities, may mask persistent or rising trends in independent stimulant toxicity, especially in the context of the “fourth wave”. While opioid-involved overdoses may respond to reversal agents, stimulant overdoses that do not involve opioids represent a distinct toxicity crisis requiring different clinical and public health responses. To address these gaps, this study utilizes the CDC Wide-ranging Online Data for Epidemiologic Research (WONDER) database to evaluate the 2024 shifts in US drug overdose mortality, with a focus on drug overdose deaths involving stimulants without opioids. We examined the intersection of age, race/ethnicity, and geographic region and their corresponding mortality patterns to uncover granular subpopulation trends that aggregate national statistics obscure.

## 2 Methods

We analyzed publicly available data on unintentional DODs from 2018 to 2024 using the CDC WONDER data. Because CDC WONDER provides de-identified, public-use data that do not involve identifiable human subjects, this study was exempt from review according to the University of Minnesota Institutional Review Board policy. All data were analyzed and reported in strict accordance with CDC data use restrictions to ensure individual privacy and data confidentiality. Drug overdose deaths were identified using underlying cause of death ICD-10 codes (Appendix Table S1). Among these deaths, substance-specific overdose deaths were then identified using the multiple cause of death ICD-10 codes for each substance (Appendix Table S2). These included T40.4 for fentanyl (which historically includes other synthetic opioids) and T43.6 for methamphetamine (which includes other psychostimulants/amphetamines), as the vast majority of deaths in these respective categories are driven by these two substances. The DODs for stimulants without opioids were calculated by subtracting opioid/stimulant co-involvement deaths from the total deaths for each respective stimulant. The population estimates used as denominators were obtained directly from the CDC WONDER platform, which are based on U.S. Census Bureau data for the same year as the DOD data. We calculated the age-adjusted mortality rates of DODs per 100,000 people, standardized to the 2000 US standard population.

To detect significant temporal changes and estimate the Annual Percent Change (APC) for each drug category from 2018 to 2024, we applied a joinpoint regression model using the “segmented” package in R (version 4.3.1). A log-linear model was first fitted with the natural logarithm of the age-adjusted mortality rate (ln(Y + 0.01)) as the dependent variable and calendar year as the independent variable; the constant 0.01 was added to handle potential zero counts. We specified one joinpoint to identify trend shifts, utilizing the Davies test to evaluate statistical significance (*p* < 0.05). For each segment, the slope coefficient (β) was estimated and converted into the APC using the formula: APC = (e^β^ − 1) × 100%.

Given the descriptive and joinpoint regression analyses showing distinct patterns for DODs involving stimulants without opioids, we performed further intersectionally stratified analysis of DODs and calculated percent change in mortality rate from 2023 to 2024 for cocaine and methamphetamine overdoses without opioids, stratified by: (1) race/ethnicity: non-Hispanic White (White), non-Hispanic Black (Black), non-Hispanic American Indian/Alaska Native (AIAN), Hispanic, and Other. However, due to the extremely small number of deaths within the ‘other’ racial/ethnic category, these data were omitted from the final intersectional stratified results to prevent unstable estimates and comply with data suppression guidelines. (2) age group: younger than 55 years (<55) and 55 years and older (55+). This age stratification was selected based on prior epidemiological evidence(Li et al., 2026) and our preliminary data inspection (Fig. S1), which indicated highly distinct overdose trend trajectories between these two cohorts. (3) census region: Midwest, Northeast, South, and West.

Age-adjusted mortality rates were calculated independently within each broad age stratum (<55 and 55+) via the direct standardization method using 10-year age intervals and the 2000 U.S. Standard Population(Anderson & Rosenberg, 1998). To evaluate the statistical significance of the mortality rate changes between 2023 and 2024 across these strata, two-sample z-tests were performed following the National Center for Health Statistics (NCHS) guidelines (Miniño, Arias, Kochanek, Murphy, & Smith, 2002).

## 3 Results

**Fig. 1:**
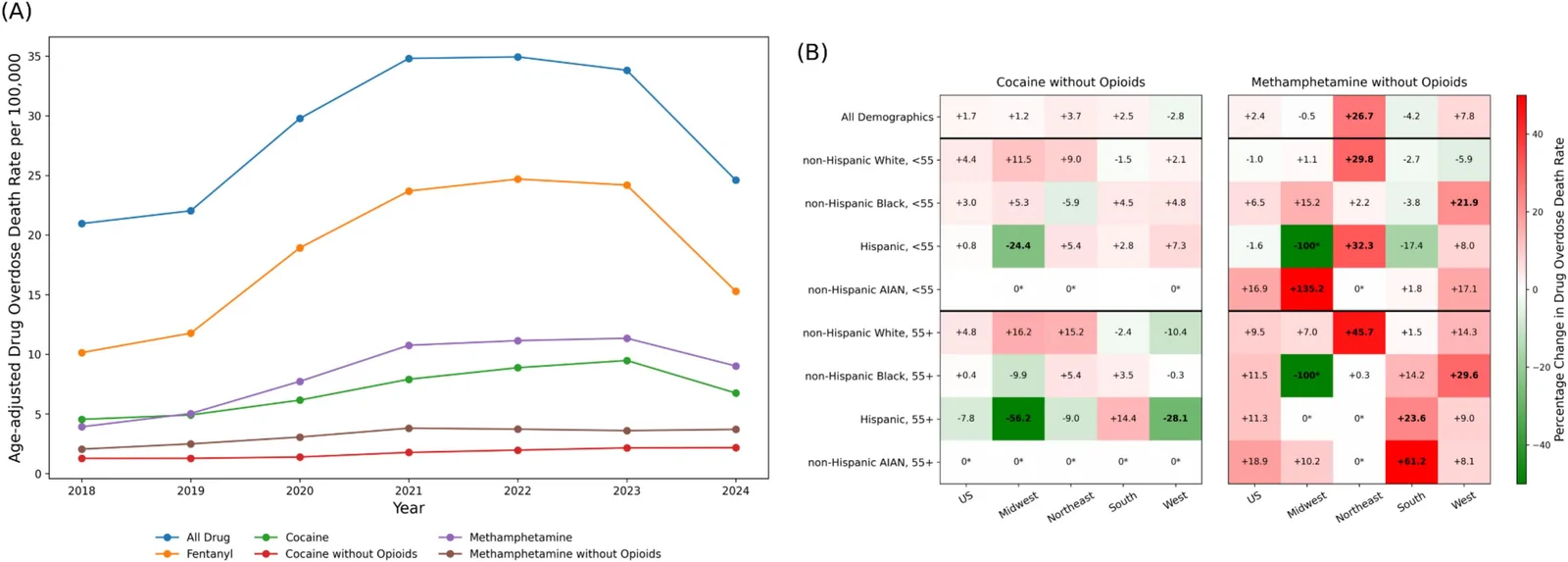
Trends in US drug overdose death rates. Panel A: Age-adjusted drug overdose death rates by substance, 2018–2024. Panel B: Percentage change in drug overdose death rates for stimulants without opioids from 2023 to 2024 by demographic group and region. In Panel B, blank cells indicate that data were suppressed due to low death counts. We chose 2023 as the comparison year based on findings from both the prior study(Post et al., 2025) and our joinpoint analysis, which identified statistically significant temporal changes for most substance types between 2022 and 2023. *Values of 0% reflect identical mortality rates in 2023 and 2024, while values of −100% arise when 2024 mortality rates were zero. Such extreme percentages should be interpreted with caution, as they often reflect low absolute death counts in specific strata.

Nationally, based on annual calendar year data, age-adjusted DOD rates peaked in 2022 (36.9 per 100,000) and decreased by 27.9% (compared to 2022) in 2024 (25.6 per 100,000), a trend that paralleled reductions in mortality involving fentanyl (38.3% reduction) (Fig. 1, Panel A). Although overall stimulant-involved mortality declined in 2024, deaths involving stimulants without opioid co-involvement continued to increase (166 [1.7%] for cocaine and 476 [2.4%] for methamphetamine; Tables S4a and S4c). Two-sample z-tests revealed that the change in national DOD rate for stimulants without opioids did not achieve statistical significance (*p* = 0.288 for cocaine; *p* = 0.080 for methamphetamine). Specifically, overdoses mortality rates for cocaine without opioids and methamphetamine without opioids exhibited stable or upward trends in 2024, which contrasted with the sharp reductions observed in overdoses involving opioids.

**Fig. 2:**
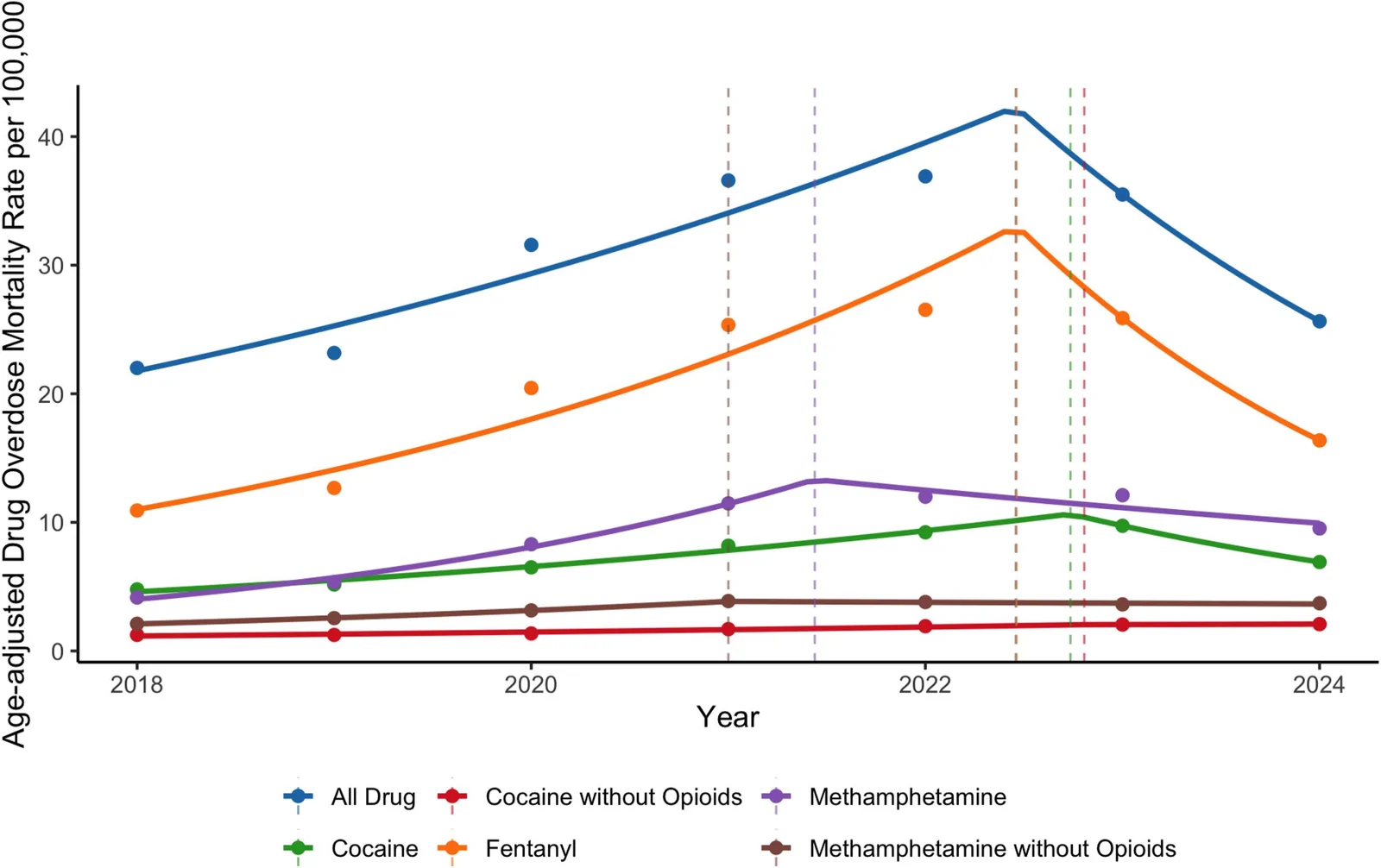
Joinpoint regression analysis of U.S. drug overdose mortality trends (2018–2024). All substance-specific mortality rates reached turning points before 2023 and began to decline thereafter. This figure illustrates the overall downward trends during 2023–2024, which are the focus of the main analysis in this study. The vertical dashed lines represent the statistically significant inflection points (joinpoints) identified by the joinpoint regression models for each respective substance category (the color indicates the substance category) indicating years where a significant change in the mortality trend occurred.

The joinpoint regression results, presented in Fig. 2 and Appendix Table S3, demonstrates that DOD rates for the majority of substance categories reached a statistical significant inflection point (trend changing point) prior to 2023, followed by a prevailing decline, including all drugs (2022−2023), fentanyl (2022–2023), cocaine (2022–2023), and methamphetamine (2021−2022). We detected a statistically significant inflection point for DOD rate for methamphetamine without opioids in 2021, followed by a milder decline after the inflection point. However, no statistically significant inflection point was identified for DOD rate for cocaine without opioids.

Fig. 1, Panel B and Table S4 display changes in DOD rate for stimulants without opioids across demographic groups and regions between 2023 and 2024. Nationwide, the DOD rate increased by 1.7% for cocaine without opioids, with larger increases seen among White individuals (<55 and 55+), while the rate declined rate among the 55+ Hispanic population. In comparison, the DOD rate increased by 2.4% for methamphetamine without opioids, with more pronounced increases observed among Black and AIAN populations and among the 55+ populations.

Regional patterns also revealed heterogeneity (Fig. 1, Panel B). In the Midwest, changes in stimulant-involved overdose deaths without opioids showed pronounced racial/ethnic divergence. DOD rates increased among White populations in both age groups for both cocaine and methamphetamine without opioids, including a 16.2% increase among 55+ individuals for cocaine without opioids. In contrast, rates declined substantially among Hispanic populations across both age groups and both stimulant types (without opioids), including a 100% reduction (these extreme percentage changes should be interpreted with caution as they likely reflect instability from small case counts) among <55 individuals for methamphetamine without opioids and a 56.2% reduction among 55+ individuals for cocaine without opioids. Patterns among Black populations diverged by age, with increases among <55 individuals (+5.3% for cocaine and + 15.2% for methamphetamine without opioids) but declines among 55+ individuals. Notably, methamphetamine without opioids overdose mortality increased by 135.2% among AIAN individuals, representing the largest subgroup increase observed nationwide.

In the Northeast, stimulant-involved DODs without opioids showed the largest overall increases across regions, with rates rising by 3.7% for cocaine without opioids and 26.7% for methamphetamine without opioids. Increases were observed among White populations in both age groups for both stimulant types, including substantial increases in methamphetamine-involved DODs without opioids among 55 + (+45.7%) and < 55 (+29.8%) White individuals. Declines were observed only for cocaine without opioids among <55 Black and 55+ Hispanic populations. Notably, methamphetamine-involved DODs without opioids also increased markedly among the <55 Hispanic population (+32.3%).

In the South, race/ethnicity-related patterns were distinct for cocaine without opioids. DOD rates increased for Black and Hispanic populations across both age groups, whereas rates declined slightly among White populations in both age groups. For methamphetamine without opioids, trends were driven more by age, where DOD rates declined among most <55 populations (except AIAN) but increased among all 55+ groups, including a 61.2% and 23.6% rise among 55+ AIAN and Hispanic populations, respectively.

In the West, DOD rates for cocaine without opioids exhibited a clear divergence by age, which generally increased among <55 populations, including a 7.3% rise for Hispanic individuals, but decreased among 55+ populations, such as a 28.1% decline for Hispanic individuals. Conversely, trends for methamphetamine without opioids showed broad increases across demographic groups, including a 21.9% and 29.6% increase for <55 and 55+ Black individuals, respectively, with the exception of the <55 White population who experienced a 5.9% decline.

## 4 Discussion

Consistent with previous studies, our findings confirm that the overall decline in US DODs is largely driven by reductions in fentanyl-involved DODs, which may be attributable to: (1) sudden shifts in the unregulated drug supply, (2) a potential “cohort effect” as high-risk populations have died, and (3) expanded overdose prevention interventions (e.g., naloxone distribution) (Garnett & Miniño, 2026; Post et al., 2025). Understanding the drivers of this decline is of major scientific and policy interest, although the underlying mechanisms remain uncertain. A recent analysis highlights that the sharp decline may be partly driven by a disruption in the illicit fentanyl supply, reflected by declining fentanyl purity, fewer seizures, and widespread reports of shortages among users. The authors hypothesize that this supply shock may be linked to increased Chinese enforcement and regulation of fentanyl precursor chemicals, which may have constrained production and reduced the potency of fentanyl entering North American drug markets (Vangelov et al., 2026). Despite the decline in overall DODs, our study finds that DODs involving stimulants without opioids continued to rise (cocaine: +1.7%; methamphetamine: +2.4%). Although these aggregate national increments are modest, they stand in contrast to the substantial declines in other drug categories. We note that the absolute rates of stimulant-without-opioid deaths remain substantially lower than those involving opioids. Because these trends are measured from a comparatively low baseline, relative increases may appear proportionally larger and may be more susceptible to year-to-year variability. Although the national increases in stimulant-without-opioid deaths did not reach statistical significance, we interpret these findings as an epidemiologically meaningful signal: their public health importance lies in the divergence from the overall downward trend and in the concentrated increases among specific subgroups. These subgroup increases were also regionally distinct and demographically disparate. The eastward expansion of methamphetamine into the Northeast suggests a homogenization of the drug supply, exposing new populations to high-potency stimulants (Ciccarone, 2021; Hoopsick, Ni, Sauda, Lee, & Yockey, 2025). Furthermore, the disproportionate increases in stimulant-involved (no opioids) DODs among Black and AIAN populations highlight the persistence of structural racism, manifesting as unequal access to treatment and housing stability, which limits the reach of current prevention efforts (Friedman, Nguemeni Tiako, & Hansen, 2024; Miles et al., 2023). Additionally, the rising rates, particularly in DODs involving methamphetamine without opioids, among 55+ adults underscore the compounded risks of potent stimulants on cardiovascular systems of this population, creating a unique clinical challenge (Chhatre, Cook, Mallik, & Jayadevappa, 2017; Curran et al., 2022; Dickson et al., 2021; Kevil et al., 2019). Possible drivers for these patterns include the increased availability and potency of stimulants, aging of the stimulant-using population coupled with elevated cardiovascular risk from long-term stimulant use, the lack of effective pharmacological treatments, and structural barriers to care, including structural racism and stigma surrounding stimulant use (Alves et al., 2023; Chang et al., 2025; Kline et al., 2023).

This study has limitations. First, it is descriptive and represents only a two-year snapshot of trends following the peak, limiting causal inference and assessment of longer-term trends; further, we did not examine the underlying drivers of these changes. Second, our reliance on CDC WONDER data means findings are subject to potential misclassification of drugs involved in overdose deaths. Specifically, because drug overdose deaths often involve multiple substances, medical certifiers (particularly in jurisdictions relying on coroners rather than medical examiners) may not record all involved drugs on the death certificate. Additionally, the analytical sample is strictly restricted to overdose deaths occurring among U.S. residents; thus, our data do not capture all overdose events that occurred within the geographic boundaries of the United States, such as those involving international tourists or short-term non-resident visitors. Furthermore, data suppression and small numbers for some demographic groups in CDC WONDER data may potentially mask underlying trends in smaller subpopulations. These suppressed or unstable estimates necessitate caution during interpretation. Finally, CDC WONDER population denominators can lag the latest intercensal or postcensal estimates. This could modestly affect estimated DOD rates, but because year-to-year population changes are generally small relative to the overdose mortality changes observed, we do not expect this limitation to materially affect our findings or conclusions.

Despite these limitations, the identified trends provide policymakers and healthcare providers with timely understanding of the evolving DOD crisis, from an opioid-dominated epidemic to a more complex landscape involving standalone stimulant use, underscoring the urgent need to expand prevention strategies beyond opioid-centric interventions to include stimulant-specific treatments. Results can help identify focal populations for new overdose prevention efforts which should include: stimulant-specific interventions, such as scaling contingency management programs (Coughlin et al., 2025), treating underlying chronic medical conditions (Chang et al., 2025), and expanding access to drug checking and safer use supplies (Syvertsen, Cabral, Knaap, Rey, & Pollini, 2025).

## Funding

The study was funded by the National Institite on Drug Abuse (NIDA), grant number: R33DA062346. NIDA had no role in the study design, collection, analysis or interpretation of the data, writing the manuscript, or the decision to submit the paper for publication.

## CRediT authorship contribution statement

**Yanwei Jin:** Writing – original draft, Visualization, Methodology, Investigation, Formal analysis, Data curation, Conceptualization. **Zongbo Li:** Writing – review & editing, Methodology, Conceptualization. **Qiyou Wu:** Writing – review & editing, Visualization, Data curation. **Brandon D.L. Marshall:** Writing – review & editing, Funding acquisition. **Erika L. Crable:** Writing – review & editing. **Xiao Zang:** Writing – review & editing, Supervision, Methodology, Funding acquisition, Conceptualization.

## Declaration of competing interest

The authors declare that they have no known competing financial interests or personal relationships that could have appeared to influence the work reported in this paper.

## Data Availability

All data are publicly available.
