# Supplementary material for: US drug overdose deaths involving stimulants without opioids continued to rise in 2024
Source: Addict Behav Rep. 2026 Aug 27;24:100743. doi: 10.1016/j.abrep.2026.100743 (PMC13545347; doi:10.1016/j.abrep.2026.100743)
Supplement: Supplementary file 1 [file mmc1.docx]

**Appendix for “US drug overdose deaths involving stimulants without opioids continued to rise in 2024”**

**1. Data sources**

**Definition of race/ethnicity:**

For race/ethnicity, we used the definition in CDC’s WONDER data (6,8). Racial/ethnic groups were defined first by ethnicity (Hispanic Origin, yes vs. no) and subsequently by race. From 1999 to 2017, race was categorized into four groups; from 2018 to 2023, six race categories were available. Based on these classifications, we defined the following mutually exclusive racial/ethnic groups: non-Hispanic White (White), non-Hispanic Black (Black), non-Hispanic American Indian or Alaska Native (AI/AN), Hispanic or Latino (any race) (Hispanic), and other race/ethnicities (i.e., Asian, Native Hawaiian or Other Pacific Islander, more than one race, and race/ethnicity not stated or available) (Other). We further stratified the first four groups (excluding Other) by age into two categories: younger than 55 years old (“<55”), and 55 years old and older (“55+”). The Other group was not stratified due to small counts. In total, nine subgroups were analyzed.

**Definition of regions:**

**Northeastern:** Connecticut, Maine, Massachusetts, New Hampshire, New Jersey, New York, Pennsylvania, Rhode Island, and Vermont

**Midwest:** Illinois, Indiana, Iowa, Kansas, Michigan, Minnesota, Missouri, Nebraska, North Dakota, Ohio, South Dakota, and Wisconsin

**South**: Alabama, Arkansas, Delaware, District of Columbia, Florida, Georgia, Kentucky, Louisiana, Maryland, Mississippi, North Carolina, Oklahoma, South Carolina, Tennessee, Texas, Virginia, and West Virginia

**West**: Alaska, Arizona, California, Colorado, Hawaii, Idaho, Montana, Nevada, New Mexico, Oregon, Utah, Washington, and Wyoming.

**Table S1. Underlying Cause of Death (UCD) ICD-10 codes**

| **ICD-10 Code** | **Description** |
| --- | --- |
| X40 | Accidental poisoning by and exposure to nonopioid analgesics, antipyretics, and antirheumatics |
| X41 | Accidental poisoning by and exposure to antiepileptic, sedative-hypnotic, antiparkinsonism, and psychotropic drugs not elsewhere classified |
| X42 | Accidental poisoning by and exposure to narcotics and psychodysleptics [hallucinogens] not elsewhere classified |
| X43 | Accidental poisoning by and exposure to other drugs acting on the autonomic nervous system |
| X44 | Accidental poisoning by and exposure to other and unspecified drugs, medicaments, and biological substances |

**Table S2.** **Substance-Specific Drug Overdose Death Codes**

| **Category** | **ICD-10 Codes** |
| --- | --- |
| Cocaine | T40.5: Poisoning by and exposure to cocaine |
| Fentanyl | T40.4: Poisoning by and exposure to synthetic opioids, excluding methadone (e.g., fentanyl, tramadol) |
| Methamphetamine | T43.6: Poisoning by and exposure to psychostimulants with abuse potential (e.g., methamphetamine) |
| Cocaine without Opioids | T40.5 and not (T40.0–T40.4 or T40.6), where opioid-related codes include:  T40.0: Poisoning by and exposure to opium  T40.1: Poisoning by and exposure to heroin  T40.2: Poisoning by and exposure to other opioids (e.g., morphine, codeine)  T40.3: Poisoning by and exposure to methadone  T40.4: Poisoning by and exposure to synthetic opioids, excluding methadone (e.g., fentanyl, tramadol)  T40.6: Poisoning by and exposure to other and unspecified narcotics |
| Methamphetamines without Opioids | T43.6 and not (T40.0–T40.4 or T40.6), where opioid-related codes are the same as listed above. |

**Table S3.** **Joinpoint regression results for U.S. drug overdose mortality trends (2018–2024)**

| **Drug** | **Breakpoint (Year)** | **p-value** | **APC before breakpoint (%)** | **APC after breakpoint (%)** | **Significance**  **(p < 0.05)** |
| --- | --- | --- | --- | --- | --- |
| All Drug | 2022.46 | 0.032 | 16.07 | -27.76 | * |
| Cocaine | 2022.74 | 0.012 | 19.35 | -28.91 | * |
| Cocaine without opioids | 2022.81 | 0.697 | 12.36 | 1.73 | ns |
| Fentanyl | 2022.46 | 0.029 | 27.98 | -36.72 | * |
| Methamphetamine | 2021.44 | 0.013 | 41.64 | -10.85 | * |
| Methamphetamine without opioids | 2021.00 | 0.004 | 22.29 | -1.78 | ** |

Notes: APC = Annual Percent Change

**Table S4a. Cocaine (without opioids) overdose deaths by demographic and region 2023-2024**

| **Demographic** | **US 2023** | **US 2024** | **MW 2023** | **MW2024** | **NE 2023** | **NE 2024** | **SO2023** | **SO2024** | **WE2023** | **WE2024** |
| --- | --- | --- | --- | --- | --- | --- | --- | --- | --- | --- |
| **Overall** |  |  |  |  |  |  |  |  |  |  |
| **All Demographics** | 5940 | 6106 | 1170 | 1182 | 1271 | 1346 | 2625 | 2722 | 874 | 856 |
| **Younger adults (<55)** | |  |  |  |  |  |  |  |  |  |
| **non-Hispanic White, <55** | 1323 | 1374 | 304 | 336 | 311 | 335 | 501 | 493 | 207 | 210 |
| **non-Hispanic Black, <55** | 949 | 1000 | 182 | 194 | 149 | 143 | 531 | 564 | 87 | 99 |
| **Hispanic, <55** | 686 | 726 | 103 | 83 | 127 | 140 | 286 | 309 | 170 | 194 |
| **non-Hispanic AIAN, <55** | suppressed | 13 | suppressed | suppressed | suppressed | suppressed | suppressed | 13 | suppressed | suppressed |
| **Older adults (55+)** | |  |  |  |  |  |  |  |  |  |
| **non-Hispanic White, 55+** | 973 | 1016 | 197 | 227 | 242 | 277 | 424 | 416 | 110 | 96 |
| **non-Hispanic Black, 55+** | 1676 | 1702 | 359 | 332 | 340 | 363 | 804 | 833 | 173 | 174 |
| **Hispanic, 55+** | 216 | 204 | 13 | 6 | 65 | 62 | 79 | 94 | 59 | 42 |
| **non-Hispanic AIAN, 55+** | suppressed | suppressed | suppressed | suppressed | suppressed | suppressed | suppressed | suppressed | suppressed | suppressed |

Note: MW = Midwest, NE = Northeast, SO = South, WE = West.

**Table S4b. Cocaine (without opioids) age-adjusted overdose mortality rate (per 100000) by demographic and region**

| **Demographic** | **US 2023** | **US 2024** | **MW 2023** | **MW2024** | **NE 2023** | **NE 2024** | **SO2023** | **SO2024** | **WE2023** | **WE2024** |
| --- | --- | --- | --- | --- | --- | --- | --- | --- | --- | --- |
| **Overall** |  |  |  |  |  |  |  |  |  |  |
| **All Demographics** | 2.05 | 2.09 | 1.97 | 2 | 2.5 | 2.59 | 2.33 | 2.39 | 1.31 | 1.28 |
| **Younger adults (<55)** | |  |  |  |  |  |  |  |  |  |
| **non-Hispanic White, <55** | 1.01 | 1.05 | 0.9 | 1 | 1.31 | 1.43* | 1.05 | 1.04 | 0.79 | 0.8 |
| **non-Hispanic Black, <55** | 3.17 | 3.26 | 3.63 | 3.82 | 3.19 | 3.01 | 3.02 | 3.16 | 3.19 | 3.35 |
| **Hispanic, <55** | 1.41 | 1.42 | 2.27 | 1.72 | 1.91 | 2.02 | 1.48 | 1.52 | 0.92 | 0.99 |
| **non-Hispanic AIAN, <55** | 0 | 0.81 | 0 | 0 | 0 | 0 | 0 | 2.37 | 0 | 0 |
| **Older adults (55+)** | |  |  |  |  |  |  |  |  |  |
| **non-Hispanic White, 55+** | 0.37 | 0.39 | 0.3 | 0.34 | 0.48 | 0.55* | 0.45 | 0.44 | 0.22 | 0.2 |
| **non-Hispanic Black, 55+** | 3.83 | 3.85 | 5.16 | 4.65 | 4.84 | 5.1 | 3.08 | 3.19 | 4.58 | 4.56 |
| **Hispanic, 55+** | 0.43 | 0.39 | 0.3 | 0.13 | 0.85 | 0.78 | 0.41 | 0.47 | 0.3 | 0.22 |
| **non-Hispanic AIAN, 55+** | 0 | 0 | 0 | 0 | 0 | 0 | 0 | 0 | 0 | 0 |

Note: MW = Midwest, NE = Northeast, SO = South, WE = West.

* Indicates a statistically significant difference between 2023 and 2024 mortality rates (p < 0.05) based on a two-sample z-test. Rates of 0.00 or based on counts <10 are suppressed or unreliable and were excluded from hypothesis testing.

**Table S4c. Methamphetamine (without opioids) overdose deaths by demographic and region 2023-2024**

| **Demographic** | **US 2023** | **US 2024** | **MW 2023** | **MW2024** | **NE 2023** | **NE 2024** | **SO2023** | **SO2024** | **WE2023** | **WE2024** |
| --- | --- | --- | --- | --- | --- | --- | --- | --- | --- | --- |
| **Overall** |  |  |  |  |  |  |  |  |  |  |
| **All Demographics** | 9927 | 10403 | 1462 | 1456 | 378 | 489 | 3942 | 3872 | 4145 | 4586 |
| **Younger adults (<55)** | |  |  |  |  |  |  |  |  |  |
| **non-Hispanic White, <55** | 3926 | 3892 | 818 | 826 | 206 | 264 | 1869 | 1831 | 1033 | 971 |
| **non-Hispanic Black, <55** | 816 | 876 | 102 | 119 | 49 | 52 | 437 | 415 | 228 | 290 |
| **Hispanic, <55** | 964 | 983 | 23 | suppressed | 21 | 31 | 285 | 244 | 635 | 708 |
| **non-Hispanic AIAN, <55** | 150 | 178 | 6 | 14 | suppressed | suppressed | 43 | 45 | 101 | 119 |
| **Older adults (55+)** | |  |  |  |  |  |  |  |  |  |
| **non-Hispanic White, 55+** | 2968 | 3224 | 457 | 485 | 91 | 131 | 1087 | 1095 | 1333 | 1513 |
| **non-Hispanic Black, 55+** | 369 | 405 | 35 | suppressed | 11 | 11 | 112 | 128 | 211 | 266 |
| **Hispanic, 55+** | 350 | 402 | suppressed | suppressed | suppressed | suppressed | 69 | 88 | 281 | 314 |
| **non-Hispanic AIAN, 55+** | 51 | 60 | 11 | 12 | suppressed | suppressed | 10 | 16 | 30 | 32 |

Note: MW = Midwest, NE = Northeast, SO = South, WE = West.

**Table S4d. Methamphetamine (without opioids) age-adjusted overdose mortality rate (per 100000) by demographic and region**

| **Demographic** | **US 2023** | **US 2024** | **MW 2023** | **MW2024** | **NE 2023** | **NE 2024** | **SO2023** | **SO2024** | **WE2023** | **WE2024** |
| --- | --- | --- | --- | --- | --- | --- | --- | --- | --- | --- |
| **Overall** |  |  |  |  |  |  |  |  |  |  |
| **All Demographics** | 3.62 | 3.71 | 2.68 | 2.67 | 0.85 | 1.08* | 3.82 | 3.65* | 6.18 | 6.66* |
| **Younger adults (<55)** | |  |  |  |  |  |  |  |  |  |
| **non-Hispanic White, <55** | 3.01 | 2.98 | 2.44 | 2.46 | 0.88 | 1.14* | 3.96 | 3.85 | 3.94 | 3.71 |
| **non-Hispanic Black, <55** | 2.68 | 2.85 | 2.01 | 2.31 | 1.02 | 1.05 | 2.41 | 2.32 | 8.38 | 10.21* |
| **Hispanic, <55** | 2 | 1.97 | 0.42 | 0 | 0.32 | 0.42 | 1.48 | 1.22* | 3.61 | 3.91 |
| **non-Hispanic AIAN, <55** | 8.85 | 10.35 | 2.28 | 5.36 | 0 | 0 | 7.72 | 7.86 | 13.04 | 15.26 |
| **Older adults (55+)** | |  |  |  |  |  |  |  |  |  |
| **non-Hispanic White, 55+** | 1.13 | 1.24* | 0.68 | 0.73 | 0.18 | 0.26* | 1.16 | 1.18 | 2.64 | 3.02* |
| **non-Hispanic Black, 55+** | 0.83 | 0.92 | 0.49 | 0 | 0.15 | 0.15 | 0.43 | 0.49 | 5.3 | 6.87* |
| **Hispanic, 55+** | 0.69 | 0.77 | 0 | 0 | 0 | 0 | 0.35 | 0.43 | 1.48 | 1.61 |
| **non-Hispanic AIAN, 55+** | 1.95 | 2.32 | 2.55 | 2.81 | 0 | 0 | 1.09 | 1.76 | 2.71 | 2.93 |

Note: MW = Midwest, NE = Northeast, SO = South, WE = West.

* Indicates a statistically significant difference between 2023 and 2024 mortality rates (p < 0.05) based on a two-sample z-test. Rates of 0.00 or based on counts <10 are suppressed or unreliable and were excluded from hypothesis testing.

**Figure S1. USA Overdose Crude Rate by Demographic (2018–2024).**

Note： The subplots illustrate a comparative analysis using three different definitions for older age groups (Top: 35+, Middle: 45+, Bottom: 55+) across various substances and combinations. Preliminary analyses indicate that while similar upward-then-downward trajectories exist across cohorts, the 55+ age group exhibits the most pronounced and distinct pattern in specific substance categories.
